# Analysis of Psychiatric Symptoms and Suicide Risk Among Younger Adults in China by Gender Identity and Sexual Orientation

**DOI:** 10.1001/jamanetworkopen.2023.2294

**Published:** 2023-03-24

**Authors:** Shufang Sun, Shicun Xu, Arryn Guy, John Guigayoma, Yanwen Zhang, Yuanyuan Wang, Don Operario, Runsen Chen

**Affiliations:** 1 Department of Behavioral and Social Sciences, Brown University School of Public Health, Providence, Rhode Island; 2 International Health Institute, Brown University School of Public Health, Providence, Rhode Island; 3 Mindfulness Center, Brown University School of Public Health, Providence, Rhode Island; 4 Northeast Asian Research Center, Jilin University, Changchun, China; 5 Department of Population, Resources and Environment, Northeast Asian Studies College, Jilin University, Changchun, China; 6 China Center for Aging Studies and Social-Economic Development, Jilin University, Changchun, China; 7 Center for Alcohol and Addictions Studies, Brown University School of Public Health, Providence, Rhode Island; 8 Vanke School of Public Health, Tsinghua University, Beijing, China; 9 Key Laboratory of Brain, Cognition and Education Sciences, Ministry of Education, China; 10 School of Psychology, Center for Studies of Psychological Application, and Guangdong Key Laboratory of Mental Health and Cognitive Science, South China Normal University, China; 11 Emory University School of Public Health, Atlanta, Georgia; 12 Institute for Healthy China, Tsinghua University, Beijing, China

## Abstract

**Importance:**

Younger adults in China who are transgender and gender nonconforming (TGNC) and lesbian, gay, and bisexual (LGB) experience high levels of minority stress (eg, stigma and bullying) and adverse mental health symptoms. However, there is a lack of research documenting mental health disparities compared with their cisgender and heterosexual peers. Studies that disaggregate TGNC and LGB individuals and are conducted in nonmetropolitan regions are also needed.

**Objectives:**

To examine whether younger adult students’ psychiatric symptoms and suicide risk vary by sexual orientation and gender identity and identify risk factors within LGB younger adults and TGNC younger adults.

**Design, Setting, and Participants:**

This cross-sectional study surveyed students at 63 universities in Jilin Province, China, between October 24 and November 18, 2021. Analyses were completed May 6, 2022.

**Exposures:**

Gender identity (TGNC vs cisgender) and sexual orientation (LGB vs heterosexual).

**Main Outcomes and Measures:**

Psychiatric outcomes included depression, anxiety, traumatic stress, and nonsuicidal self-injury (NSSI), which were measured by the Patient Health Questionnaire-9, Generalized Anxiety Disorder-7, Trauma Screening Questionnaire, and self-reported NSSI behaviors. The Suicide Behaviors Questionnaire–Revised was used to assess suicide risk.

**Results:**

The analytic sample included 89 342 younger adults (mean [SD] age, 19.60 [1.75] years), including 51 438 assigned female at birth and 37 904 assigned male at birth. The sample included 2352 individuals who were TGNC, 6501 who were cisgender LGB, and 80 489 who were cisgender heterosexual. Compared with their cisgender heterosexual peers, TGNC and LGB younger adults were more likely to experience increased psychiatric symptoms across categories and suicide risk, as well as more severe forms of symptoms. In particular, overall suicide risk was 43.03% among TGNC individuals, 36.21% among LGB individuals, and 11.70% among cisgender heterosexual younger adults. Controlling for demographic variables and compared with cisgender heterosexual peers, TGNC young adults had higher odds of suicide risk (adjusted odds ratio, 5.38; 95% CI, 4.94-5.86; *P* < .001) and of past-year NSSI (adjusted odds ratio, 6.55; 95% CI, 5.87-7.30; *P* < .001). Separate within-group analyses for LGB and TGNC younger adults highlighted the vulnerability of bisexual women and gender nonbinary groups, as well as the relevance of minority stress-related risk factors, including peer bullying and assault and loneliness.

**Conclusions and Relevance:**

This study noted disparities of mental health issues among LGB, transgender, or queer or questioning younger adults in China and suggests a high urgency to address psychological health and prevent suicide in this population. Targeted public health initiatives may be needed to reduce stigma and discrimination, train competent health care professionals, and create affirmative mental health policies and systems of care.

## Key Points

**Question:**

Do transgender and gender-nonconforming (TGNC) and lesbian, gay, and bisexual (LGB) younger adults in China experience worse mental health and suicide risk than their cisgender heterosexual peers, and what are risk factors within TGNC and LGB groups?

**Findings:**

In this cross-sectional survey of 89 342 younger adults in China, TGNC and cisgender LGB individuals had a higher prevalence of depression, anxiety, traumatic stress, nonsuicidal self-injury, and suicide risk compared with cisgender heterosexual individuals. Sexual minority women, gender nonbinary individuals, and those who reported peer bullying and assault and loneliness were particularly vulnerable.

**Meaning:**

The findings of this study suggest there is an imperative need to improve mental health and prevent suicide among Chinese LGB, transgender, or queer or questioning younger adults.

## Teaser

This cross-sectional study examines psychiatric outcomes among transgender and gender-nonconforming younger adults as well as lesbian, gay, and bisexual younger adults in China.

## Introduction

Mental health is a major public health issue in China.^1^ Common mental health issues, such as depression and anxiety, have been increasing in the past decade^2^ and are especially high among adolescents, younger adults, and university students.^3^ In addition, suicide remains the leading cause of death among individuals aged 15 to 34 years, accounting for 19% of deaths.^4^ In parallel, there has been increasing attention to the mental health of lesbian, gay, and bisexual (LGB) and transgender and gender-nonconforming (TGNC) individuals globally, particularly in low- and middle-income countries and areas with high stigma contexts, such as in China.^5^ People in China who are lesbian, gay, bisexual, transgender, or queer or questioning (LGBTQ) remain hidden, and most conceal their identity.^6^ Import of early Western psychiatry led to homosexuality being categorized as a mental illness until 2000.^7^ Despite litigation against conversion therapy, it is still a visible and common practice provided in leading hospitals in major Chinese cities.^8,9^ Notably, LGBTQ individuals in China experience prevalent mental health concerns, such as depression, anxiety, and suicidal ideation.^6,8,10^ Theory-based research with gay and bisexual men in China suggests that minority stress (eg, bullying, social isolation) may be a key component of mental health issues.^6,11^ Young adulthood is a vulnerable period for mental health problems,^12^ and this period may mark heightened risks for LGBTQ individuals due to issues related to school bullying, increased salience of identity, and increased familial and societal pressure to marry and procreate. Thus, it is crucial to understand the mental health of LGBTQ younger adults and contributing factors.

There are many limitations to current mental health initiatives and research with LGBTQ people in China. First, much of the work has taken place in metropolitan areas, with a lack of data from less economically developed regions. Second, extant research with LGBTQ individuals in China has largely used within-group study designs to identify health risk factors within sexual or gender minority groups. The lack of comparison with their cisgender heterosexual peers makes it difficult to document population-level mental health disparities faced by LGBTQ groups. Furthermore, current efforts have also mainly focused on gay and bisexual men due in part to HIV-related funding influence. In addition, there is a dearth of research disaggregating TGNC individuals and their cisgender LGB peers.

To advance the evidence base on mental health disparities among LGBTQ younger adults in China, this study analyzed data from a recently completed survey of younger adults enrolled in college/university in Jilin Province, a largely industrial setting in the northeast region of China. The current study aimed to evaluate the prevalence of mental health issues, including depression, anxiety, traumatic stress, nonsuicidal self-injury (NSSI), and suicide risk, among younger adults by their sexual orientation and gender identity and identify demographic and psychological characteristics associated with psychiatric symptoms and suicide risk within samples of cisgender LGB and TGNC younger adults.

## Methods

### Recruitment and Analytic Sample

Reporting in this study is in accordance with the Strengthening the Reporting of Observational Studies in Epidemiology (STROBE) reporting guideline for observational studies. University students in Jilin Province, China, were recruited for a cross-sectional online survey. Students from all 63 universities in Jilin were invited to participate in the survey. Recruitment was conducted through universities’ staff members and targeted advertisements (eg, WeChat student groups). After reading the study information, interested individuals were invited to scan a quick response code that brought them to the study informed consent form and the secure, anonymous questionnaire. Participants provided consent by selecting the commensurate button. Individuals who completed the survey were eligible to enter a lottery for cash prizes by clicking on a different link. The study was approved by the ethics committee in Jilin University. Data were collected between October 24 and November 18, 2021. Data analysis was completed May 6, 2022.

A total of 117 769 individuals consented to participate in the survey. Among them, a total of 23 701 were excluded due to failed attention checks (correction rate <75%, n = 21 541), invalid data (n = 10), inconsistent responses on gender identity questions (eg, selected male sex at birth and transgender male on separate questions, selected transgender on 1 question and checked cisgender on another question; n = 2150). Furthermore, as the current study focused on TGNC and LGB individuals, non-TGNC gender minority individuals (eg, “cross-dresser” or selected “unsure about gender identity”), those who identified as asexual or unsure about their sexual orientation, and those who did not provide valid gender identity or sexual orientation responses were excluded from the analysis (n = 4726). The final analytic sample comprised 89 342 individuals.

### Measures

Demographic information included participants’ age, ethnicity (Han or non-Han), educational level, residence of origin (city or rural/suburban), whether they are the only child, and family’s income level. Consistent with practices suggested by the Williams Institute,^13^ the following questions assessed participants’ gender identity. First, participants were asked, “What sex were you assigned at birth? (the sex showing on your original birth certificate),” with choices of male and female. Next, participants were asked, “If you could only choose one option, which of the following best describes you?” Options were “man,” “woman,” “trans man,” “trans woman,” “genderqueer/gender nonbinary/gender nonconforming,” “cross-dresser,” “unsure,” and “not listed (fill-in text).” Transgender individuals included those who were assigned female at birth (AFAB) and chose male or trans man on the second question and those who were assigned male at birth (AMAB) and chose female or trans woman on the second question. For those who reported “not listed” to the gender identity question, 2 graduate research assistants reviewed participants’ responses and coded gender identity based on the fill-in text provided. Furthermore, participants were asked a third question earlier in the survey, “What is your gender identity (ie, your understanding on your gender),” with options including cisgender, transgender, genderqueer/gender nonbinary/gender nonconforming, unsure, and don’t understand the question. Given the cultural context and potential for low familiarity with these terms, we compared the first 2 questions and used the third question as a validity check. Only TGNC individuals who answered consistently across these questions were included in the analysis.

Regarding sexual orientation, participants were asked, “What best describes your sexual orientation?” Options included asexual, gay/lesbian, heterosexual, bisexual/pansexual, and not listed (fill-in text). For those who responded “not listed,” 2 research assistants further coded participants’ sexual orientation after manual review of their fill-in text responses, their gender identity, and participants’ responses to 2 additional questions on sexual and romantic attractions. Thus, all participants were categorized as TGNC, cisgender LGB, or cisgender heterosexual for analysis. The eFigure in Supplement 1 provides a flowchart on gender and sexual orientation coding of the sample in detail.

Two psychosocial vulnerability variables included peer bullying and assault and loneliness. Participants indicated yes or no on whether they had experienced peer bullying in the past year on the following types: verbal bullying, physical violence, sexual harassment, and internet-based bullying and assault. Loneliness was assessed by the 3-item UCLA Loneliness Scale.^14^

### Psychiatric Symptoms and Suicide Risk

Psychiatric outcome variables included depression, anxiety, traumatic stress, and NSSI. The Patient Health Questionnaire-9 measured depressive symptoms,^15^ which reflects the 9 *Diagnostic and Statistical Manual of Mental Disorders* (Fourth Edition) criteria for a major depressive episode,^16^ with higher scores indicating more severe symptoms. The Generalized Anxiety Disorder-7 (Chinese version) assessed anxiety symptoms,^17^ with higher scores representing more severe symptoms. The Trauma Screening Questionnaire (Chinese version) assessed traumatic stress,^18^ which has 10 items designed to detect posttraumatic stress disorder (PTSD) symptoms. Participants self-reported NSSI behaviors through 2 questions that were adapted based on the Clinician Rated Severity of Non-Suicidal Self-Injury.^16,19^ Participants first indicated if they had ever engaged in NSSI (>1 year ago, within the past year, or never); if they engaged in recent NSSI (<1 year ago), they were asked to indicate the number of days.

The Suicide Behaviors Questionnaire–Revised assessed suicide risk with 4 items that asked lifetime suicidal ideation, frequency of suicidal ideation in the past 12 months, threat of suicide attempt, and self-reported likelihood of suicide attempt.^20^ A cutoff of total score greater than or equal to 7 indicates suicide risk in adult outpatient populations.^20^

### Statistical Analysis

We estimated unadjusted frequencies of demographic characteristics for subsamples of TGNC individuals, cisgender LGB individuals, and cisgender heterosexual individuals. Prevalence rates of depression, anxiety, traumatic stress, NSSI, and suicide risk in these 3 subsamples were calculated. Next, through performing logistic regression, we calculated adjusted odds ratios (aORs) and 95% CIs of LGBTQ status compared with their cisgender heterosexual peers associated with psychiatric and suicide outcomes (depression, anxiety, traumatic stress, NSSI, and suicide risk) after adjusting for demographic variables (age, sex assigned at birth, ethnicity, family income, residence, and only-child status). In addition, 2 sets of logistic regression analyses were conducted among the cisgender LGB sample and TGNC sample to estimate aORs and 95% CIs for demographic and social factors associated with psychiatric symptoms and suicide risk in these 2 samples. All null hypothesis significance testing was conducted with a 2-tailed α level of significance of .05. Data analysis was performed in R, version 2022.07.1 (R Foundation for Statistical Computing) and relevant packages (psych, dplyr).

## Results

### Demographic and Social Characteristics

The analytic sample of 89 342 individuals (51 438 AFAB and 37 904 AMAB) included 2352 TGNC individuals, 6501 cisgender LGB individuals, and 80 489 cisgender heterosexual individuals; mean (SD) age was 19.60 (1.75) years. Table 1 presents demographic characteristics of the sample by their gender identity and sexual orientation (TGNC, cisgender LGB, and cisgender heterosexual individuals), as well as their between-group comparisons (cisgender LGB vs cisgender heterosexual and TGNC vs cisgender individuals). Compared with their heterosexual peers, more LGB younger adults were AFAB (74.56% LGB vs 55.79% heterosexual), from an urban region (61.07% LGB vs 49.77% heterosexual), and the only child (51.95% LGB vs 47.00% heterosexual). Compared with cisgender younger adults, more TGNC participants were AFAB (71.60% TGNC vs 55.79% cisgender), non-Han ethnicity (12.16% TGNC vs 10.32% cisgender), and from urban areas (56.72% TGNC vs 49.77% cisgender). The LGB and TGNC younger adults also reported significantly higher frequencies of peer bullying and assault. All forms of bullying are reported in Table 1.

**Table 1.** Sample Demographic Characteristics of 89 342 Participants

Variable	Cisgender heterosexual, No. (%) (n=80 489)	LGBT samples, No. (%)		*P* value	
		Cisgender LGB (n=6501)	TGNC (n=2352)	Cisgender LGB vs cisgender heterosexual^a^	TGNC vs cisgender^b^
Age, mean (SD), y	19.60 (1.75)	19.56 (1.69)	19.58 (1.87)	.06	.76
Sex at birth					
Male	35 582 (44.21)	1654 (25.44)	668 (28.40)	<.001	<.001
Female	44 907 (55.79)	4847 (74.56)	1684 (71.60)		
Ethnicity					
Han	72 179 (89.68)	5784 (88.98)	2066 (87.84)	.09	.006
Other	8310 (10.32)	717 (11.02)	286 (12.16)		
Home residence					
Urban	40 060 (49.77)	3970 (61.07)	1334 (56.72)	<.001	<.001
Suburban/rural	40 429 (50.23)	2531 (38.93)	1018 (43.28)		
Home region^c^					
China					
North	9328 (11.59)	681 (10.48)	254 (10.80)	<.001	<.001
Northeast	47 686 (59.25)	3525 (54.22)	1385 (58.89)		
East	8940 (11.11)	860 (13.23)	239 (10.16)		
South Central	6842 (8.50)	738 (11.35)	219 (9.31)		
Southwest	3985 (4.95)	428 (6.58)	156 (6.63)		
Northwest	3699 (4.60)	267 (4.11)	97 (4.12)		
Hong Kong or Macau	9 (0.01)	2 (0.03)	2 (0.09)		
Annual family income, Yuan					
<6000	24 020 (29.84)	1559 (23.98)	728 (30.95)	<.001	.60
>6000-14 000	26 135 (32.47)	2093 (32.20)	738 (31.38)		
>14 000-23 000	13 399 (16.65)	1210 (18.61)	396 (16.84)		
>23 000-36 000	7894 (9.81)	722 (11.11)	226 (9.61)		
>36 000-70 000	5383 (6.69)	564 (8.68)	151 (6.42)		
>70 000	3658 (4.54)	353 (5.43)	113 (4.80)		
Only-child status					
Only child	37 829 (47.00)	3377 (51.95)	1129 (48.00)	<.001	.56
Have sibling	42 660 (53.00)	3124 (48.05)	1223 (52.00)		
Education (currently pursuing)					
Undergraduate degree	78 144 (97.10)	6327 (97.34)	2281 (97.02)	.55	.39
Master’s degree	2219 (2.76)	166 (2.55)	69 (2.93)		
Doctorate degree	115 (0.14)	7 (0.11)	1 (0.04)		
Peer bullying					
Any	4975 (6.18)	742 (11.41)	404 (17.18)	<.001	<.001
Verbal	4272 (5.31)	601 (9.24)	333 (14.16)	<.001	<.001
Physical assault	822 (1.02)	102 (1.57)	64 (2.72)	<.001	<.001
Sexual harassment	327 (0.41)	113 (1.74)	54 (2.30)	<.001	<.001
Cyber	711 (0.88)	133 (2.05)	83 (3.53)	<.001	<.001
Loneliness					
Not lonely	59 517 (73.94)	3929 (60.44)	1270 (54.00)	<.001	<.001
Lonely	20 972 (26.06)	2572 (39.56)	1082 (46.00)	<.001	<.001

Abbreviations: LGB, lesbian, gay, and bisexual; LGBT, lesbian, gay, bisexual, and transgender; TGNC, transgender and gender nonconforming.

^a^ *P* value determined using *t* test or χ^2^ test comparing cisgender LGB sample with cisgender heterosexual sample.

^b^ *P* value determined using *t* test or χ^2^ test comparing TGNC with cisgender samples (heterosexual and LGB).

^c^ For home region, North China includes Beijing, Tianjin, Hebei, Shanxi, and Inner Mongolia; Northeast China includes Liaoning, Jilin, and Heilongjiang; East China includes Shanghai, Jiangsu, Zhejiang, Anhui, Fujian, Jiangxi, and Shandong; South Central China includes Henan, Hubei, Hunan, Guangdong, Guangxi, and Hainan; Southwest China includes Chongqing, Sichuan, Guizhou, Yunan, and Tibet; and Northwest China includes Shaanxi, Gansu, Qinghai, Ningxia, and Xinjiang.

### Psychiatric Symptoms and Suicide Risk

As reported in Table 2, compared with their heterosexual and cisgender peers, TGNC and cisgender LGB younger adults were more likely to experience depression, anxiety, traumatic stress, NSSI, and suicide risk. A total of 66.51% LGB, 70.96% TGNC, and 47.29% cisgender heterosexual younger adults had clinically elevated depressive symptoms (mild or more severe). Furthermore, 51.75% LGB, 56.93% TGNC, and 33.85% cisgender heterosexual younger adults had elevated anxiety symptoms. As reported in Table 2, LGB and TGNC individuals were also more likely to report more severe forms of depressive and anxiety symptoms compared with their cisgender heterosexual peers. Approximately one-fifth of the full sample (18.77%) reported traumatic stress, including 17.23% cisgender heterosexual, 30.27% cisgender LGB, and 39.41% TGNC younger adults. Regarding NSSI, cisgender LGB and TGNC individuals reported a higher proportion of a history of NSSI (20.6% LGB and 27.42% TGNC vs 6.25% cisgender heterosexual), as well as recent NSSI (<1 year: 13.17% LGB and 19.73% TGNC vs 3.46% cisgender heterosexual). The prevalence of overall suicide risk was 11.70% among cisgender heterosexual younger adults, 36.21% among LGB individuals, and 43.03% among TGNC individuals. Specifically, 6.45% LGB, 9.01% TGNC, and 1.78% cisgender heterosexual younger adults made a suicide attempt in their lifetime; 53.7% LGB, 57.27% TGNC, and 23.79% cisgender heterosexual younger adults had thought about attempting suicide in the past year and 19.61% LGB, 25.55% TGNC, and 5.61% cisgender heterosexual individuals had a suicide plan at least once.

**Table 2.** Psychiatric Symptoms and Suicide Risk Among 89 342 Participants^a^

Variable	No. (%)		
	Cisgender heterosexual	Cisgender LGB	TGNC
No.	80 489	6501	2352
Depression, symptoms^b^			
None (0-4)	42 425 (52.71)	2177 (33.49)	683 (29.04)
Mild (5-9)	30 299 (37.64)	2934 (45.13)	971 (41.28)
Moderate (10-14)	5542 (6.89)	907 (13.95)	387 (16.45)
Moderately severe (15-19)	1529 (1.90)	328 (5.05)	190 (8.08)
Severe (20-27)	694 (0.86)	155 (2.38)	121 (5.14)
Anxiety, symptoms^c^			
None (0-4)	53 244 (66.15)	3137 (48.25)	1013 (43.07)
Mild (5-9)	22 968 (28.54)	2533 (38.96)	895 (38.05)
Moderate (10-14)	3078 (3.82)	577 (8.88)	273 (11.61)
Severe (≥15)	1199 (1.49)	254 (3.91)	171 (7.27)
Traumatic stress			
Presence of PTSD (≥6)^d^	13 872 (17.23)	1968 (30.27)	927 (39.41)
NSSI			
None	75 460 (93.75)	5162 (79.40)	1707 (72.58)
>1 y Ago	2243 (2.79)	483 (7.43)	181 (7.70)
Recent (<1 y, 1-4 d)	1642 (2.04)	435 (6.69)	223 (9.48)
Recent (<1 y, ≥5 d)	1144 (1.42)	421 (6.48)	241 (10.25)
Suicide risk			
Overall (≥7)^e^	9416 (11.70)	2354 (36.21)	1012 (43.03)
Attempt, yes	1430 (1.78)	419 (6.45)	212 (9.01)
Ideation, yes	19 145 (23.79)	3489 (53.67)	1347 (57.27)
Plan, yes	4512 (5.61)	1275 (19.61)	601 (25.55)

Abbreviations: LGB, lesbian, gay, and bisexual; NSSI, nonsuicidal self-harm; PTSD, posttraumatic stress disorder; TGNC, transgender and gender nonconforming.

^a^ *P* values determined with *t* test or χ^2^ test comparing cisgender LGB sample with cisgender heterosexual sample and TGNC with cisgender samples (heterosexual and LGB). All differences are significant at *P* < .001.

^b^ Scored using the Patient Health Questionnaire-9.

^c^ Scored using the Generalized Anxiety Disorder-7.

^d^ Scored using the Trauma Screening Questionnaire.

^e^ Scored using the Suicide Behaviors Questionnaire–Revised.

Controlling for demographic characteristics, Table 3 reports the aORs of psychiatric symptoms and suicide risk outcomes among LGBTQ participants, with cisgender heterosexual younger adults as the reference group. Cisgender LGB and TGNC individuals had elevated risks across all outcomes. Specifically, compared with their cisgender heterosexual peers, cisgender LGB younger adults were at increased odds of reporting clinically elevated depression (aOR, 2.22; 95% CI, 2.11-2.34; *P* < .001), anxiety (aOR, 2.07; 95% CI, 1.97-2.18; *P* < .001), traumatic stress (aOR, 2.11; 95% CI, 2.00-2.23; *P* < .001), history of NSSI (aOR, 3.73; 95% CI, 3.49-3.99; *P* < .001), recent NSSI (aOR, 3.97; 95% CI, 3.65-4.31; *P* < .001), overall suicide risk (aOR, 3.99; 95% CI, 3.77-4.22; *P* < .001), lifetime suicide attempt (aOR, 3.55; 95% CI, 3.16-3.97; *P* < .001), suicidal ideation in the past 12 months (aOR, 3.46; 95% CI, 3.28-3.64; *P* < .001), and suicide plan in the past (aOR, 3.83; 95% CI, 3.58-4.11; *P* < .001). Similarly, compared with cisgender heterosexual younger adults, TGNC participants were at increased odds of experiencing depression (aOR, 2.70; 95% CI, 2.47-2.96; *P* < .001), anxiety (aOR, 2.54; 95% CI, 2.34-2.77; *P* < .001), traumatic stress (aOR, 3.14; 95% CI, 2.88-3.42; *P* < .001), lifetime NSSI history (aOR, 5.52; 95% CI, 5.01-6.07; *P* < .001), recent NSSI (aOR, 6.55; 95% CI, 5.87-7.30; *P* < .001), overall suicide risk (aOR, 5.38; 95% CI, 4.94-5.86; *P* < .001), lifetime suicide attempt (aOR, 5.16; 95% CI, 4.43, 5.99; *P* < .001), suicidal ideation in the past 12 months (aOR, 4.07; 95% CI, 3.74-4.43; *P* < .001), and suicide plan in the past (aOR, 5.51; 95% CI, 4.99-6.07; *P* < .001).

**Table 3.** TGNC and Cisgender LGB Status Compared With Cisgender Heterosexual Peers With Psychiatric Symptoms and Suicide Risk^a^

Dependent variable	TGNC	Cisgender LGB
	aOR (95% CI)	aOR (95% CI)
Depression	2.70 (2.47-2.96)	2.22 (2.11-2.34)
Anxiety	2.54 (2.34-2.77)	2.07 (1.97-2.18)
PTSD	3.14 (2.88-3.42)	2.11 (2.00-2.23)
Ever NSSI	5.52 (5.01-6.07)	3.73 (3.49-3.99)
Recent NSSI (<1 y)	6.55 (5.87-7.30)	3.97 (3.65-4.31)
Suicide risk		
Overall	5.38 (4.94-5.86)	3.99 (3.77-4.22)
Attempt	5.16 (4.43-5.99)	3.55 (3.16-3.97)
Ideation	4.07 (3.74-4.43)	3.46 (3.28-3.64)
Plan	5.51 (4.99-6.07)	3.83 (3.58-4.11)

Abbreviations: aOR, adjusted odds ratio; LGB, lesbian, gay, and bisexual; NSSI, nonsuicidal self-harm; PTSD, posttraumatic stress disorder; TGNC, transgender and gender nonconforming.

^a^ Age, sex at birth, ethnicity, family income, residence (rural or suburban), and only-child status were controlled in all models. All differences are significant at *P* < .001.

### Demographic and Psychosocial Factors Associated With Psychiatric Symptoms and Suicide Risk Among Cisgender LGB Younger Adults

Among LGB younger adults, compared to their gay men peers, being a bisexual woman was associated with a higher risk of depression (aOR, 1.41; 95% CI, 1.15-1.72; *P* < .001), anxiety (aOR, 1.23; 95% CI, 1.02-1.49; *P* < .001), NSSI (aOR, 1.71; 95% CI, 1.35-2.19; *P* < .001 for NSSI history and aOR, 2.26; 95% CI, 1.66-3.15; *P* < .001 for recent NSSI), and suicide risk (aOR, 1.81; 95% CI, 1.49-2.22; *P* < .001); being a lesbian woman was associated with a higher risk of NSSI (aOR, 1.74; 95% CI, 1.23-2.46; *P* = .002 for NSSI history and aOR, 2.28; 95% CI, 1.48-3.53; *P* < .001 for recent NSSI). Being from a rural region was associated with a lower risk of NSSI (aOR, 0.81; 95% CI, 0.70-0.93; *P* = .002 for NSSI history and aOR, 0.83; 95% CI, 0.71-0.98; *P* = .03 for recent NSSI) and suicide (aOR, 0.87; 95% CI, 0.78-0.98; *P* = .02). Only-child status was associated with a lessened risk for depression (aOR, 0.88; 95% CI, 0.78-0.98; *P* = .02) and PTSD (aOR, 0.86; 95% CI, 0.77-0.97; *P* = .02), yet it was a risk factor for NSSI (aOR, 1.15; 95% CI, 1.01-1.31; *P* = .04 for NSSI history and aOR, 1.23; 95% CI, 1.05-1.44; *P* = .009 for recent NSSI). Across all psychiatric outcomes, peer bullying and assault (aOR, 2.00; 95% CI, 1.63-2.48; *P* < .001 for depression; aOR, 1.79; 95% CI, 1.50-2.13; *P* < .001 for anxiety; aOR, 2.25; 95% CI, 1.91-2.65; *P* < .001 for PTSD; aOR, 1.70; 95% CI, 1.43-2.02; *P* < .001 for NSSI history; aOR, 1.95; 95% CI, 1.60-2.36; *P* < .001 for recent NSSI; and aOR, 1.83; 95% CI, 1.55-2.15; *P* < .001 for suicide risk) and loneliness (aOR, 4.36; 95% CI, 3.85-4.96; *P* < .001 for depression; aOR, 3.92; 95% CI, 3.51-4.37; *P* < .001 for anxiety; aOR, 4.04; 95% CI, 3.61-4.54; *P* < .001 for PTSD; aOR, 2.63; 95% CI, 2.32-2.99; *P* < .001 for NSSI history; aOR, 2.85; 95% CI, 2.45-3.33; *P* < .001 for recent NSSI; and aOR, 3.13; 95% CI, 2.81-3.49; *P* < .001 for suicide risk) were associated with higher risk of all outcomes. Table 4 reports aORs and their CIs.

**Table 4.** Demographic and Psychosocial Factors Associated With Psychiatric Symptoms and Suicide Risk Among 6501 Cisgender LGB Young Adults

Independent variable	Depression		Anxiety		PTSD		NSSI				Suicide risk	
							Ever		Recent			
	aOR (95% CI)	*P* value	aOR (95% CI)	*P* value	aOR (95% CI)	*P* value	aOR (95% CI)	*P* value	aOR (95% CI)	*P* value	aOR (95% CI)	*P* value
Sexual orientation												
Men												
Gay	1 [Reference]	NA	1 [Reference]	NA	1 [Reference]	NA	1 [Reference]	NA	1 [Reference]	NA	1 [Reference]	NA
Bisexual	1.07 (0.85-1.34)	.56	1.11 (0.89-1.38)	.37	0.98 (0.78-1.24)	.88	0.96 (0.72-1.28)	.77	1.16 (0.81-1.70)	.42	0.91 (0.72-1.15)	.43
Women												
Bisexual	1.41 (1.15-1.72)	<.001	1.23 (1.02-1.49)	.03	0.99 (0.81-1.21)	.90	1.71 (1.35-2.19)	<.001	2.26 (1.66-3.15)	<.001	1.81 (1.49-2.22)	<.001
Lesbian	1.11 (0.83-1.49)	.46	0.99 (0.75-1.31)	.95	0.97 (0.71-1.32)	.84	1.74 (1.23-2.46)	.002	2.28 (1.48-3.53)	<.001	1.31 (0.97-1.77)	.08
Age	0.97 (0.94-1.00)	.09	0.96 (0.94-0.99)	.02	0.99 (0.96-1.03)	.74	1.01 (0.98-1.05)	.52	1.02 (0.98-1.07)	.36	0.99 (0.96-1.02)	.55
Family income	0.99 (0.95-1.03)	.64	1.01 (0.98-1.05)	.43	1.03 (0.99-1.07)	.19	1.01 (0.96-1.05)	.72	1.00 (0.95-1.05)	.92	0.98 (0.94-1.01)	.20
Ethnicity												
Han	1 [Reference]	NA	1 [Reference]	NA	1 [Reference]	NA	1 [Reference]	NA	1 [Reference]	NA	1 [Reference]	NA
Non-Han	1.01 (0.85-1.21)	.88	1.05 (0.89-1.24)	.59	1.17 (0.98-1.40)	.07	0.99 (0.81-1.20)	.90	0.98 (0.77-1.24)	.89	1.12 (0.94-1.32)	.20
Home residence												
Urban	1 [Reference]	NA	1 [Reference]	NA	1 [Reference]	NA	1 [Reference]	NA	1 [Reference]	NA	1 [Reference]	NA
Rural	1.04 (0.92-1.17)	.50	0.97 (0.86-1.08)	.58	0.92 (0.81-1.04)	.20	0.81 (0.70-0.93)	.002	0.83 (0.71-0.98)	.03	0.87 (0.78-0.98)	.02
Only-child status												
Have siblings	1 [Reference]	NA	1 [Reference]	NA	1 [Reference]	NA	1 [Reference]	NA	1 [Reference]	NA	1 [Reference]	NA
Only child	0.88 (0.78-0.98)	.02	0.91 (0.82-1.02)	.10	0.86 (0.77-0.97)	.02	1.15 (1.01-1.31)	.04	1.23 (1.05-1.44)	.009	0.97 (0.87-1.09)	.63
Peer bullying and assault												
Not subjected to	1 [Reference]	NA	1 [Reference]	NA	1 [Reference]	NA	1 [Reference]	NA	1 [Reference]	NA	1 [Reference]	NA
Subjected to	2.00 (1.63-2.48)	<.001	1.79 (1.50-2.13)	<.001	2.25 (1.91-2.65)	<.001	1.70 (1.43-2.02)	<.001	1.95 (1.60-2.36)	<.001	1.83 (1.55-2.15)	<.001
Loneliness												
Not lonely	1 [Reference]	NA	1 [Reference]	NA	1 [Reference]	NA	1 [Reference]	NA	1 [Reference]	NA	1 [Reference]	NA
Lonely	4.36 (3.85-4.96)	<.001	3.92 (3.51-4.37)	<.001	4.04 (3.61-4.54)	<.001	2.63 (2.32-2.99)	<.001	2.85 (2.45-3.33)	<.001	3.13 (2.81-3.49)	<.001

Abbreviations: aOR, adjusted odds ratio; LGB, lesbian, gay, and bisexual; NA, not applicable; NSSI, nonsuicidal self-harm; PTSD, posttraumatic stress disorder.

### Demographic and Psychosocial Factors Associated With Psychiatric Symptoms and Suicide Risk Among TGNC Younger Adults

In the subsample of TGNC younger adults, being nonbinary-AFAB (aOR, 1.65; 95% CI, 1.22-2.22; *P* = .001 for depression; aOR, 1.40; 95% CI, 1.06-1.85; *P* = .02 for anxiety; aOR, 1.57; 95% CI, 1.18-2.08; *P* = .002 for PTSD; aOR, 2.44; 95% CI, 1.82-3.30; *P* < .001 for NSSI history; aOR, 2.91; 95% CI, 2.09-4.07; *P* < .001 for recent NSSI; and aOR, 3.13; 95% CI, 2.37-4.16; *P* < .001 for suicide risk, as compared to their transgender women peers), peer bullying and assault (aOR, 1.99; 95% CI, 1.45-2.77; *P* < .001 for depression; aOR, 2.30; 95% CI, 1.76-3.02; *P* < .001 for anxiety; aOR, 2.95; 95% CI, 2.32-3.77; *P* < .001 for PTSD; aOR, 2.48; 95% CI, 1.96-3.15; *P* < .001 for NSSI history; aOR, 2.63; 95% CI, 2.05-3.38; *P* < .001 for recent NSSI; and aOR, 2.41; 95% CI, 1.89-3.07; *P* < .001 for suicide risk), and loneliness (aOR, 5.93; 95% CI, 4.75-7.44; *P* < .001 for depression; aOR, 4.57; 95% CI, 3.81-5.51; *P* < .001 for anxiety; aOR, 4.16; 95% CI, 3.46-5.00; *P* < .001 for PTSD; aOR, 2.80; 95% CI, 2.29-3.42; *P* < .001 for NSSI history; aOR, 3.08; 95% CI, 2.45-3.88; *P* < .001 for recent NSSI; and aOR, 3.48; 95% CI, 2.90-4.17; *P* < .001 for suicide risk) were associated with increased risk for all psychiatric outcomes. Being nonbinary-AMAB and trans men were associated with an increased risk for depression (aOR, 1.96; 95% CI, 1.19-3.32; *P* = .01 among nonbinary-AMAB and aOR, 1.33; 95% CI, 1.05-1.69; *P* = .02 among trans men), NSSI history (aOR, 2.19; 95% CI, 1.42-3.37; *P* < .001 among nonbinary-AMAB and aOR, 1.42; 95% CI, 1.10-1.84; *P* = .01 among trans men), recent NSSI (aOR, 1.89; 95% CI, 1.16-3.05; *P* = .01 among nonbinary-AMAB and aOR, 1.41; 95% CI, 1.06-1.91; *P* = .02 among trans men), and suicide risk (aOR, 3.37; 95% CI, 2.21-5.18; *P* < .001 among nonbinary-AMAB and aOR, 1.64; 95% CI, 1.30-2.07; *P* < .001 among trans men). The only-child status was associated with an increased risk for NSSI. Table 5 presents detailed aORs and CIs.

**Table 5.** Demographic and Psychosocial Factors Associated With Psychiatric Symptoms and Suicide Risk Among 2352 TGNC Young Adults

Independent variable	Depression		Anxiety		PTSD		NSSI				Suicide risk	
							Ever		Recent			
	aOR (95% CI)	*P* value	aOR (95% CI)	*P* value	aOR (95% CI)	*P* value	aOR (95% CI)	*P* value	aOR (95% CI)	*P* value	aOR (95% CI)	*P* value
Gender identity												
Trans women	1 [Reference]	NA	1 [Reference]	NA	1 [Reference]		1 [Reference]		1 [Reference]		1 [Reference]	
Nonbinary												
AFAB	1.65 (1.22-2.22)	.001	1.40 (1.06-1.85)	.02	1.57 (1.18-2.08)	.002	2.44 (1.82-3.30)	<.001	2.91 (2.09-4.07)	<.001	3.13 (2.37-4.16)	<.001
AMAB	1.96 (1.19-3.32)	.01	1.46 (0.95-2.28)	.09	1.40 (0.92-2.13)	.12	2.19 (1.42-3.37)	<.001	1.89 (1.16-3.05)	.01	3.37 (2.21-5.18)	<.001
Trans men	1.33 (1.05-1.69)	.02	1.03 (0.82-1.29)	.78	1.01 (0.80-1.28)	.90	1.42 (1.10-1.84)	.01	1.41 (1.06-1.91)	.02	1.64 (1.30-2.07)	<.001
Age	0.98 (0.93-1.03)	.49	0.95 (0.91-1.00)	.06	0.99 (0.94-1.04)	.66	0.97 (0.92-1.03)	.35	0.98 (0.92-1.04)	.45	0.99 (0.94-1.04)	.66
Family Income	0.98 (0.91-1.05)	.54	0.97 (0.91-1.04)	.39	0.99 (0.92-1.05)	.69	1.01 (0.94-1.08)	.75	1.00 (0.92-1.08)	.94	0.97 (0.91-1.04)	.45
Ethnicity												
Han	1 [Reference]	NA	1 [Reference]	NA	1 [Reference]	NA	1 [Reference]	NA	1 [Reference]	NA	1 [Reference]	NA
Non-Han	1.17 (0.87-1.59)	.31	1.16 (0.88-1.53)	.28	1.21 (0.92-1.59)	.18	0.83 (0.61-1.11)	.22	0.87 (0.61-1.21)	.41	0.88 (0.67-1.16)	.37
Home residence												
Urban	1 [Reference]	NA	1 [Reference]	NA	1 [Reference]	NA	1 [Reference]	NA	1 [Reference]	NA	1 [Reference]	NA
Rural	1.08 (0.88-1.34)	.45	1.10 (0.91-1.33)	.33	0.90 (0.74-1.09)	.29	0.82 (0.67-1.02)	.07	0.80 (0.63-1.01)	.06	0.89 (0.74-1.08)	.24
Only-child status												
Have siblings	1 [Reference]	NA	1 [Reference]	NA	1 [Reference]	NA	1 [Reference]	NA	1 [Reference]	NA	1 [Reference]	NA
Only child	1.08 (0.88-1.32)	.46	1.04 (0.86-1.25)	.71	0.88 (0.73-1.07)	.20	1.41 (1.15-1.72)	<.001	1.29 (1.03-1.62)	.03	1.08 (0.90-1.30)	.42
Peer bullying and assault												
Not subjected to	1 [Reference]	NA	1 [Reference]	NA	1 [Reference]	NA	1 [Reference]	NA	1 [Reference]	NA	1 [Reference]	NA
Subjected to	1.99 (1.45-2.77)	<.001	2.30 (1.76-3.02)	<.001	2.95 (2.32-3.77)	<.001	2.48 (1.96-3.15)	<.001	2.63 (2.05-3.38)	<.001	2.41 (1.89-3.07)	<.001
Loneliness												
Not lonely	1 [Reference]	NA	1 [Reference]	NA	1 [Reference]	NA	1 [Reference]	NA	1 [Reference]	NA	1 [Reference]	NA
Lonely	5.93 (4.75-7.44)	<.001	4.57 (3.81-5.51)	<.001	4.16 (3.46-5.00)	<.001	2.80 (2.29-3.42)	<.001	3.08 (2.45-3.88)	<.001	3.48 (2.90-4.17)	<.001

Abbreviations: AFAB, assigned female at birth; AMAB, assigned male at birth; aOR, adjusted odds ratio; NA, not applicable; NSSI, nonsuicidal self-harm; PTSD, posttraumatic stress disorder; TGNC, transgender and gender-nonconforming.

## Discussion

To our knowledge, this is the largest cross-sectional study that examined psychiatric symptoms among younger adults by gender identity and sexual orientation in China and the first that highlights the disparities of mental health and suicide risk for TGNC and LGB younger adults compared with their cisgender heterosexual peers. Moreover, this study underscores the relevance of minority stress-related psychosocial factors (ie, bullying, assault, and loneliness) as contributors to adverse mental health symptoms among Chinese TGNC and LGB younger adults. These findings are important to possibly guide efforts on LGBTQ mental health promotion in China, particularly in university settings.

Overall, across gender identity and sexual orientation categories, younger adults reported prevalent psychiatric symptoms and suicidal ideation, with observed rates being higher than findings from previous meta-analyses in this group.^21,22^ This may reflect increasing mental health issues among younger adults in China due to a combination of adverse societal factors, including the COVID-19 pandemic, economic recession, and increased competition in the work force.^23^ However, compared with their cisgender heterosexual peers, TGNC and LGB younger adults had heightened prevalence and more severe symptoms across all outcomes. In particular, high suicide risk disparities warrant urgent public health interventions designed for LGBTQ younger adults in China.

There is insufficient research in China and other low- and middle-income country contexts to understand the heterogeneity and subgroup experiences within the LGBTQ community, particularly among bisexual and gender nonbinary people.^24,25^ Bisexual women and gender nonbinary younger adults in our sample, particularly transmasculine individuals, had higher risks compared with their other LGB and TGNC peers across many outcomes. As the current research among LGBTQ people in China largely focuses on gay and bisexual men, study findings call for greater visibility and awareness of mental health needs of sexual minority women and gender minority individuals, especially transmasculine and nonbinary people. Significant barriers impede TGNC individuals from receiving gender-affirmative medical care. The guideline for gender-affirming surgery by the National Health Commission requires patients to receive a diagnosis of gender dysphoria or transsexualism from their psychiatrist.^26^ As gender-affirmative medical care is essential to reduce the burden of mental health issues in this group, mental health professionals serve as important gatekeepers for the health of TGNC individuals in China. Our findings suggest an urgent need for the medical community in China to stop considering diverse gender identities as pathological and to develop gender-affirming guidelines on mental health care with TGNC individuals. In this regard, increasing the number of mental health counselors on college/university campuses, providing training on LGBTQ-affirmative therapy, and enhancing health care professionals’ advocacy skills can be fruitful.

Study findings also noted a significant level of peer bullying and assault and loneliness in the mental health of TGNC and LGB younger adults. Consequently, university-level initiatives are needed to create more LGBTQ-friendly environments and reduce isolation among LGBTQ students. University health care systems—from primary care to psychological health centers—may need to work together to develop LGBTQ-targeted mental health and suicide prevention and intervention programs.

Being the only child was consistently a risk factor for NSSI across LGB and TGNC younger adults. A recent meta-analysis on NSSI among Chinese youths suggests adverse life events and problematic parent-child relationship are both contributing factors,^27^ and it is possible that for LGBTQ younger adults in China, only-child status was a risk factor for NSSI in part attributed to potential parental rejection and lack of other support sources. It is unclear why being from rural areas was associated with a lower risk of NSSI and suicide among LGB individuals and warrants further understanding through research on relevant factors (eg, outness, peer bullying and assault, and school/city climate).

### Limitations

The study has several limitations. First, the cross-sectional design prohibits causal inference. Second, although younger adults in this sample came from all regions of China (Table 1), as the study was conducted in Jilin, results may not be representative at a nationwide scale. Similarly, because the study was conducted among university students, findings may not be generalized to nonstudent populations. Third, LGBTQ younger adults AFAB are overrepresented in the sample. Fourth, given that the survey did not specifically target LGBTQ individuals and Chinese younger adults may be less familiar with LGBTQ identity terms, we took the conversative approach and only included those who responded consistently to gender identity questions. Future research may inquire how Chinese younger adults understand gender identity to reach consensus on how to best categorize gender minority individuals. Methodologic innovations, such as allowing for selection of multiple gender identities, may be explored in further qualitative or mixed-methods research with Chinese LGBTQ-identifying and non–LGBTQ-identifying people. As gender minority individuals also have diverse sexual orientation/identities, future research may examine the intersection of gender identity and sexual orientation in relation to mental health disparities. Fifth, whereas a substantial amount of younger adults (9.4%) identified as sexual and/or gender minority in this sample, this may represent an underestimated proportion due to the stigmatizing social-cultural environment in which data were collected and potential hesitations for self-identification as sexual and/or gender minority in an online survey. Sixth, since we did not perform false discovery adjustment, we caution against any conclusions on findings with *P* values >.001 and encourage interpretations based on 95% CIs.^28^ Seventh, clinician-rated measures are preferrable for accurate assessment of psychiatric symptoms. The self-report nature of the study makes it vulnerable to known biases, such as social desirability.

## Conclusions

Globally, gender and sexual minority youths are at higher risk for mental health issues and suicide risk compared with their cisgender heterosexual peers.^29,30,31^ Investigating such disparities in high-stigma low- and middle-income country contexts is particularly important to understand the magnitude of mental health inequality. This large survey study noted a high prevalence and disparities of mental health issues among younger adults in China by sexual orientation and gender identity. The high suicide risks among TGNC and LGB younger adults are particularly alarming. Public health initiatives may be useful to address the mental health needs and prevent suicide among LGBTQ younger adults in China, including developing and implementing LGBTQ-affirmative care in mental health and medical settings, promoting an inclusive campus climate, and fostering greater belonging and societal acceptance of LGBTQ communities.

## Notes

Supplement 1. **eFigure.** Participant Flowchart

Supplement 2.
Data Sharing Statement
