# Supplementary material for: Analysis of Psychiatric Symptoms and Suicide Risk Among Younger Adults in China by Gender Identity and Sexual Orientation
Source: JAMA Netw Open. 2023 Mar 24;6(3):e232294. doi: 10.1001/jamanetworkopen.2023.2294 (PMC10313143; doi:10.1001/jamanetworkopen.2023.2294)
Supplement: Supplement 1. — eFigure. Participant Flowchart [file jamanetwopen-e232294-s001.pdf]

Supplementary Online Content

Sun S, Xu S, Guy A, et al. Assessment of psychiatric symptoms and suicide risk among younger adults in China by gender identity and sexual orientation. *JAMA Netw Open*. 2023;6(3):e232294. doi:10.1001/jamanetworkopen.2023.2294

eFigure. Participant Flowchart

This supplementary material has been provided by the authors to give readers additional information about their work.

© 2023 Sun S et al. *JAMA Network Open.*

eFigure. Participant Flowchart

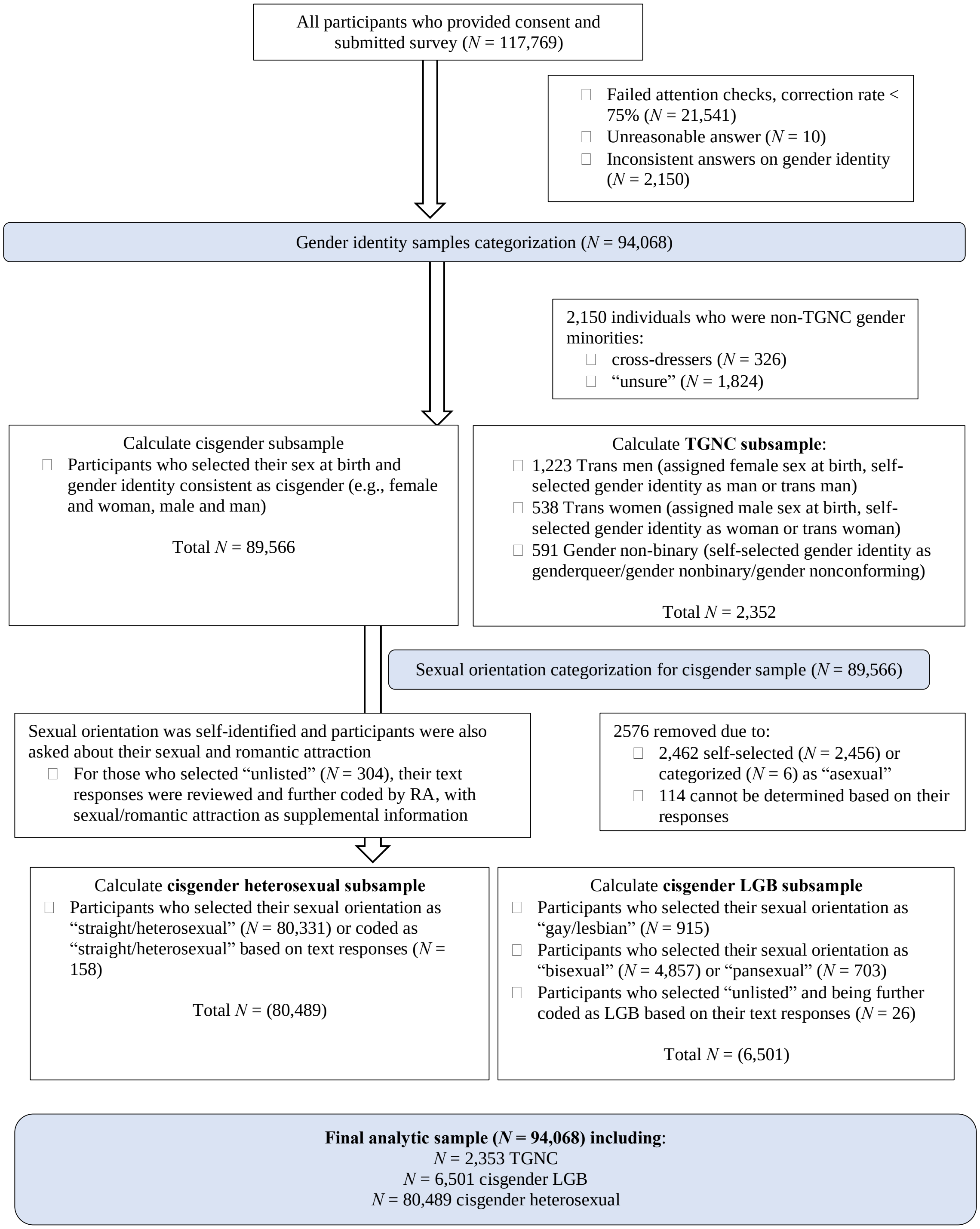

Note. As the concept of "asexuality" is less familiar to the Chinese public and misunderstanding about asexuality as a sexual identity is common (e.g., being interpreted as not sexually active) in part due to its translation as "无性恋 (non-sex love)", we took a methodologically conservative approach to focus on LGB individuals.

© 2023 Sun S et al. *JAMA Network Open.*
