# Supplementary material for: Analysis of Psychiatric Symptoms and Suicide Risk Among Younger Adults in China by Gender Identity and Sexual Orientation
Source: JAMA Netw Open. 2023 Mar 24;6(3):e232294. doi: 10.1001/jamanetworkopen.2023.2294 (PMC10313143; doi:10.1001/jamanetworkopen.2023.2294)
Supplement: Supplement 2. — Data Sharing Statement [file jamanetwopen-e232294-s002.pdf]

# Data Sharing Statement

Sun. Analysis of Psychiatric Symptoms and Suicide Risk Among Younger Adults in China by Gender Identity and Sexual Orientation. *JAMA Netw Open*. Published March 24, 2023. doi:10.1001/jamanetworkopen.2023.2294

**Data**
**Data available:** No
